# Ultradifferentiable regularity properties of a class of hypoelliptic operators

**DOI:** 10.1007/s11565-026-00758-6

**Published:** 2026-09-18

**Authors:** Stefan Fürdös

**Affiliations:** Institute of Mathematics, University of Vienna, Oskar-Morgenstern-Platz 1, 1090 Wien, Austria

**Keywords:** Ultradifferentiable hypoellipticity, Theorem of Iterates, Primary 35B65, Secondary 35H10, 35A17, 26E10

## Abstract

We investigate the ultradifferentiable regularity of solutions and vectors of a class of hypoelliptic differential operators first introduced by Hörmander. Our results extend results of Hashimoto–Morimoto–Matsuzawa and Bolley–Camus–Metivier.

## Introduction

The question of regularity of vectors of differential operators, commonly referred to as the problem of iterates, has recently experienced a surge in interest, see e.g. [7–9, 13, 15] or [16]. The survey [5] gives an overview of the older literature and the connection of the problem of iterates with the problem of hypoellipticity.

We recall that a smooth function $f\in \mathcal {E}(\Omega )$ defined in an open set $\Omega \subseteq \mathbb {R}^n$ is in the Gevrey class $\mathcal {G}^s(\Omega )$ of order s≥1 (The case s=1 corresponds to the class of real-analytic functions) if for all compact sets $K\subseteq \Omega$ there are constants C,h>0 such that

$$\begin{aligned} \sup _{x\in K}|D^\alpha f(x){|}\le Ch^{{|\alpha |}}({{{|\alpha |}}}!)^s \end{aligned}$$

for all $\alpha \in \mathbb {N}_0^n$. If *P* is a differential operator of order *d* with real-analytic coefficients on Ω then a distribution $u\in \mathcal {D}^\prime (\Omega )$ is an *s*-Gevrey vector, s≥1, if $P^k u\in L^{1}_{loc}(\Omega )$ for all $k\in \mathbb {N}_0$ and for all compact $K\subseteq \Omega$ there are constants C,h>0 such that

$$\begin{aligned} {\left\| {P^k u }\right\| }_{L^{1}(K)}\le Ch^k ((dk)!)^s \end{aligned}$$

for all $k\in \mathbb {N}_0$. The space of *s*-Gevrey vectors of *P* is denoted by $\mathcal {G}^s(\Omega ;P)$.

If *P* is an elliptic differential operator with real-analytic coefficients on an open set $\Omega \subseteq \mathbb {R}^n$ then $\mathcal {G}^s(\Omega ;P)=\mathcal {G}^s(\Omega )$, cf. [3] (for s=1 see also [18] and [20]). According to [21] the converse is also true for s>1. On the other hand it was shown in [1] for hypoelliptic differential operators *P* of principal type that $\mathcal {G}^1(\Omega ;P)=\mathcal {G}^1(\Omega )$ and there is a loss of regularity for $u\in \mathcal {G}^s(\Omega ,P)$. Similar results were proven by Derridj, see [8], for sum of squares of real vector fields of finite type.

This brief note can be viewed as a continuation of the recent paper [12] of Schindl and the author, where a novel approach to the problem of iterates was introduced. In particular, we proved, [12, Theorem 1.1], that for hypoelliptic operators of principal type an ultradifferentiable theorem of iterates hold with respect to certain ultradifferentiable classes given by weight functions in the sense of Braun–Meise–Taylor [6]. We focus our study in this paper on a class of hypoelliptic differential operators which were originally introduced by Hörmander, for a definition see the next section. In [2] Bolley–Camus investigated the Gevrey hypoellipticity and the regularity of Gevrey vectors of these operators. Their results on the Gevrey hypoellipticity were improved by Hashimoto–Morimoto–Matsuzawa [14], whereas Bolley–Camus–Metivier [4] sharpened the results on the regularity of Gevrey vectors.

We will analyze the hypoellipticity and regularity of vectors of operators in this class in the general ultradifferentiable setting given by weight matrices [22] which generalize the setting of weight sequences, cf. e.g. [19], and the classes given by weight functions, see [6]. In particular, we will prove the theorem of iterates for these operators for the same family of Braun–Meise–Taylor classes which have been considered in [12, Theorem 1.1]. Note that by [13, Remark 2.8] an analogous statement cannot hold for Denjoy–Carleman classes.

## Statement of main results

We focus in this article on a class of linear partial differential operators *P*(*x*, *D*) of some given order *d*, with real-analytic coefficients in some open set Ω, whose symbols p(x,ξ) satisfy the following conditions:

1. There are constants ρ and δ such that $0\le \delta <\rho \le 1$ and for all compact $K\subset \Omega$ there are constants C,A>0 and M>0 such that
  $$\begin{aligned} |\partial ^\alpha _{\xi }\partial ^\beta _{x} p(x,\xi ){|}\le CA^{{|\alpha |}+{|\beta |}}{|\alpha |}!{|\beta |}! |p(x,\xi ){|}{|\xi |}^{-\rho {|\alpha |}+\delta {|\beta |}} \end{aligned}$$
  for x∈K, $$\xi \in \mathbb {R}^n$$ with $${|\xi |}\ge M$$, $$\alpha ,\beta \in \mathbb {N}_0^n$$.
2. There exists some $d^\prime >0$ and for all compact subsets $\Omega \subseteq K$ there are constants C>0 and L>0 such that
  $$\begin{aligned} {|\xi |}^{d^\prime }\le C|p(x,\xi ){|} \end{aligned}$$
  for x∈K, $$\xi \in \mathbb {R}^n$$ with $${|\xi |}\ge L$$.

We denote the space of operators satisfying conditions (1) and (2) by $\mathcal {A}^{d,d^\prime }_{\rho ,\delta }(\Omega )$.

As noted above we want to discuss the ultradifferentiable hypoellipticity of operators in $\mathcal {A}^{d,d^\prime }_{\rho ,\delta }(\Omega )$. In the literature there are mainly two closely related families of ultradifferentiable classes: first, Denjoy–Carleman classes with weight sequences as defining data, and Braun–Meise–Taylor classes given by weight functions.

### Definition 2.1

A weight sequence is a sequence $\textbf{M}=(M_k)_k$ of positive real numbers such that $M_0=1$ and

$$\begin{aligned} M_k^2\le M_{k-1}M_{k+1},\qquad k\in \mathbb {N}. \end{aligned}$$

A function $f\in \mathcal {E}(\Omega )$ is ultradifferentiable of class $\{\textbf{M}\}$, respective of class $(\textbf{M})$, if for all compact sets $K\subseteq \Omega$ there are constants C,h>0, respective for all h>0 there exists C>0, such that

$$\begin{aligned} \sup _{x\in K}|D^\alpha f(x){|}\le Ch^{{|\alpha |}}M_{{|\alpha |}},\qquad \alpha \in \mathbb {N}_0^n. \end{aligned}$$

The spaces of functions of class $\{\textbf{M}\}$ and $(\textbf{M})$ are denoted by $\mathcal {E}^{\{ \textbf{M} \}} ( \Omega )$ and $\mathcal {E}^{( \textbf{M} )} ( \Omega )$, respectively.

### Definition 2.2

A weight function is a continuous, increasing function $\omega :[0,\infty )\rightarrow [0,\infty )$ such that $\omega _{[0,1]}\equiv 0$ and the following conditions are satisfied:

$$\begin{aligned} \omega (2t)=O(\omega (t))\qquad t\longrightarrow \infty , \\ \log t=o(\omega (t))\qquad t\rightarrow \infty , \\ \varphi _\omega (t):=\omega (e^t)\text { is convex.} \end{aligned}$$

If we consider also the conjugate function of $\varphi _\omega$ given by $\varphi ^*(t)=\sup _{s\ge 0}(st-\varphi (s))$ then a smooth function $f\in \mathcal {E}(\Omega )$ is of class $\{\omega \}$ if for all compact sets $K\subseteq \Omega$ there are constants C,h>0 such that

2.1 $$\begin{aligned} \sup _{x\in K} |D^\alpha f(x){|}\le C e^{h^{-1}\varphi ^*_\omega (h{|\alpha |})}\qquad \;\,\forall \,\alpha \in \mathbb {N}_0^n. \end{aligned}$$

On the other hand *f* is of class (ω) if for all compact $K\subseteq \Omega$ and every h>0 there exists C>0 such that the estimate (2.1) is satisfied.

In the following we will use the notation $[*]=\{*\},(*)$ where $*=\omega ,\textbf{M}$. If *P* is a differential operator then we say that *P* is [∗]-hypoelliptic if ${{\,\mathrm{sing\, supp}\,}}_{[*]}Pu={{\,\mathrm{sing\, supp}\,}}_{[*]} u$ for every $u\in \mathcal {D}^\prime (\Omega )$. Here ${{\,\mathrm{sing\, supp}\,}}_{[*]}u$ is the singular support of *u* with respect to the class $\mathcal {E}^{[ * ]} ( \Omega )$, which is defined in the obvious way.

### Definition 2.3

A weight sequence $\textbf{M}$ is semiregular if $\root k \of {M_k/k!}\rightarrow \infty$ for $k\rightarrow \infty$ and

$$\begin{aligned} \sup _{k\in \mathbb {N}}\root k \of {\frac{M_{k}}{M_{k-1}}}<\infty . \end{aligned}$$

An example of a semiregular weight sequence is the Gevrey sequence $\textbf{G}^\sigma =(k!^\sigma )_k$ of index $\sigma \ge 1$. We are now able to formulate the main statements of this paper:

### Theorem 2.4

Let $P\in \mathcal {A}^{d,d^\prime }_{\rho ,\delta }(\Omega )$ and $\theta =(\rho -\delta )^{-1}$. Then the following statements hold:

1. *P* is $\{\textbf{M}\}$-hypoelliptic for any semiregular weight sequence $\textbf{M}$ such that $\sup _k k^\theta /\Lambda _k<\infty$ where $\Lambda _k=\root k \of {M_k}$ is the quotient sequence associated to $\textbf{M}$.
2. *P* is $(\textbf{M})$-hypoelliptic for any semiregular weight sequence $\textbf{M}$ with $\lim _{k\rightarrow \infty }\tfrac{k^\theta }{\Lambda _k}=0$.
3. *P* is $\{\omega \}$-hypoelliptic for every weight function ω with $\omega (t)=O(t^{\rho -\delta })$, $t\rightarrow \infty$.
4. *P* is (ω)-hypoelliptic for every weight function ω such that $\omega (t)=o(t^{\rho -\delta })$, $t\rightarrow \infty$.

If *P* is a differential operator with analytic coefficients of order *d* and ω a weight function then a distribution $u\in \mathcal {D}^\prime (\Omega )$ is an element of $\mathcal {E}^{\{ \omega \}} \left( \Omega ; P \right)$ if $P^ku\in L^1_{loc}(\Omega )$ for all $k\in \mathbb {N}_0$ and for every $V\Subset \Omega$ there are C,h>0 such that

2.2 $$\begin{aligned} {\left\| {P^k u}\right\| }_{L^1(V)}\le C e^{1/h\varphi ^*_\omega (hdk)}\qquad \;\,\forall \,k\in \mathbb {N}_0. \end{aligned}$$

On the other hand, *u* is an element of $\mathcal {E}^{ (\omega ) } ( \Omega ; P )$ if $P^k u\in L^{1}_{loc}(\Omega )$ for all $k\in \mathbb {N}_0$ and for every $V\Subset \Omega$ and all h>0 there is C>0 such that (2.2) is satisfied.

### Theorem 2.5

Let ω be a weight function such that

$\Xi $ $$\begin{aligned} \;\,\exists \,H\ge 1:\quad \omega \bigl (t^2\bigr )=O(\omega (Ht)),\qquad t\longrightarrow \infty . \end{aligned}$$

Then for any $P\in \mathcal {A}_{\rho ,\delta }^{d,d^\prime }(\Omega )$ we have that

$$\begin{aligned} \mathcal {E}^{\{ \omega \}} \left( \Omega ; P \right)&=\mathcal {E}^{\{ \omega \}} ( \Omega ),\\ \mathcal {E}^{ (\omega ) } ( \Omega ; P )&=\mathcal {E}^{( \omega )} ( \Omega ). \end{aligned}$$

## Ultradifferentiable hypoellipticity

If $\textbf{M}$ and $\textbf{N}$ are two weight sequences then we set

$$\begin{aligned} \textbf{M}&\le \textbf{N}  &   :\Longleftrightarrow&M_k&\le N_k&\;\,\forall \,k&\in \mathbb {N}_0,\\ \textbf{M}&\preceq \textbf{N}  &   :\Longleftrightarrow&\;\,\exists \,C,h>0:\quad M_k&\le Ch^k N_k&\;\,\forall \,k&\in \mathbb {N}_0,\\ \end{aligned}$$

and

$$\begin{aligned} \textbf{M}&\lhd \textbf{N}  &   :\Longleftrightarrow&\;\,\forall \,h>0,\,\;\,\exists \,C>0:\quad M_k&\le Ch^k N_k&\;\,\forall \,k&\in \mathbb {N}_0. \end{aligned}$$

### Definition 3.1

A weight matrix $\mathfrak {M}$ is a family of weight sequences such that for each pair $\textbf{M},\textbf{N}\in \mathfrak {M}$ we have either $\textbf{M}\le \textbf{N}$ or $\textbf{N}\le \textbf{M}$.

Let $\Omega \subseteq \mathbb {R}^n$ be an open set and $\mathfrak {M}$ a weight matrix. A smooth function $f\in \mathcal {E}(\Omega )$ is ultradifferentiable of class $\{\mathfrak {M}\}$ if for each compact set $K\subseteq \Omega$ there are constants C,h>0 and some $\textbf{M}\in \mathfrak {M}$ such that

3.1 $$\begin{aligned} \sup _{x\in K}|D^\alpha f(x){|}\le Ch^{{|\alpha |}}M_{{|\alpha |}},\qquad \;\,\forall \,\alpha \in \mathbb {N}_0^n. \end{aligned}$$

We say that *f* is of class $(\mathfrak {M})$ if for each compact set $K\subseteq \Omega$ and every h>0 and $\textbf{M}\in \mathfrak {M}$ there is a constant C>0 such that (3.1) is satisfied. We write $\mathcal {E}^{[ \mathfrak {M} ]} ( \Omega )$ for the space of functions of class $[\mathfrak {M}]$.

If $\mathfrak {M}$ and $\mathfrak {N}$ are two weight matrices then we write $\mathfrak {M}\{\preceq \}\mathfrak {N}$ if for all $\textbf{M}\in \mathfrak {M}$ there is some $\textbf{N}\in \mathfrak {N}$ such that $\textbf{M}\preceq \textbf{N}$ and $\mathfrak {M}(\preceq )\mathfrak {N}$ if for all $\textbf{N}\in \mathfrak {N}$ there is $\textbf{M}\in \mathfrak {M}$ such that $\textbf{M}\preceq \textbf{N}$. Moreover, $\mathfrak {M}\{\lhd )\textbf{N}$ if $\textbf{M}\lhd \textbf{N}$ for all $\textbf{M}\in \mathfrak {M}$ and all $\textbf{N}\in \mathfrak {N}$. Then $\mathfrak {M}[\preceq ]\mathfrak {N}$ implies that $\mathcal {E}^{[ \mathfrak {M} ]} ( \Omega )\subseteq \mathcal {E}^{[ \mathfrak {N} ]} ( \Omega )$ whereas $\mathcal {E}^{\{ \mathfrak {M} \}} ( \Omega )\subseteq \mathcal {E}^{( \mathfrak {N} )} ( \Omega )$ when $\mathfrak {M}\{\lhd )\mathfrak {N}$.

### Definition 3.2

Let $\mathfrak {M}$ be a weight matrix.

1. We say that $\mathfrak {M}$ is R-semiregular if the following conditions hold:
  3.2 $$\begin{aligned}  &   \;\,\forall \,\textbf{M}\in \mathfrak {M}\quad \lim _{k\rightarrow \infty }\root k \of {\frac{M_k}{k!}}=\infty \end{aligned}$$
  3.3 $$\begin{aligned}  &   \;\,\forall \,\textbf{M}\in \mathfrak {M}\;\,\exists \,\textbf{N}\in \mathfrak {M}\;\,\exists \,Q>0\quad M_{k+1}\le Q^{k+1}N_k\qquad \;\,\forall \,k\in \mathbb {N}_0. \end{aligned}$$
2. The weight matrix $\mathfrak {M}$ is B-semiregular if (3.2) and
  3.4 $$\begin{aligned} \;\,\forall \,\textbf{N}\in \mathfrak {M}\;\,\exists \,\textbf{M}\in \mathfrak {M}\;\,\exists \,Q>0\quad M_{k+1}\le Q^{k+1}N_k\qquad \;\,\forall \,k\in \mathbb {N}_0 \end{aligned}$$
  hold.

### Remark 3.3

 

1. Condition (3.2) means that $\mathcal {G}^1(\Omega ) \subsetneq \mathcal {E}^{[\mathfrak {M}]}$.
2. Condition (3.3) (resp. (3.4)) implies that the Roumieu class $\mathcal {E}^{\{ \mathfrak {M} \}} ( \Omega )$ (resp. the Beurling class $\mathcal {E}^{( \mathfrak {M} )} ( \Omega )$) is closed under derivation.
3. In fact, if $\mathfrak {M}$ is [semiregular] then $\mathcal {E}^{[ \mathfrak {M} ]} ( \Omega )$ is closed under composition with real-analytic mappings: Let $\Phi :\Omega _1\rightarrow \Omega _2$ be a real-analytic mapping then $f\circ \Phi \in \mathcal {E}^{[ \mathfrak {M} ]} ( \Omega _1 )$ for all $f\in \mathcal {E}^{[ \mathfrak {M} ]} ( \Omega _2 )$.

We are going to need the following characterization of $\mathcal {E}^{[ \mathfrak {M} ]} ( \Omega )$ from [11].

### Theorem 3.4

([11, Proposition 5.1]) Let $u\in \mathcal {D}^\prime (\Omega )$, $\textbf{M}$ be a weight sequence and $p_0\in \Omega$. Then the following holds:

1. If $\mathfrak {M}$ is R-semiregular then *u* is of class $\{\mathfrak {M}\}$ near $p_0$ if and only if for some neighborhood *V* of $p_0$ there is a bounded sequence $u_k\in \mathcal {E}^\prime (\Omega )$ such that $u_k\vert _V=u\vert _V$ and there are a constant h>0 and some $\textbf{M}\in \mathfrak {M}$ so that
  3.5 $$\begin{aligned} \sup _{\begin{array}{c} \xi \in \mathbb {R}^n\\ k\in \mathbb {N}_0 \end{array}}\frac{{|\xi |}^k|\hat{u}_k(\xi ){|}}{h^kM_k}<\infty . \end{aligned}$$
2. If $\mathfrak {M}$ is B-semiregular then *u* is of class $(\mathfrak {M})$ near $p_0$ if and only if for some neighborhood *V* of $p_0$ there is a bounded sequence $u_k\in \mathcal {E}^\prime (\Omega )$ such that $u_k\vert _V=u\vert _V$ and (3.5) holds for all h>0 and every $\textbf{M}\in \mathfrak {M}$.

For the definition and theory of the wavefront set ${{\,\textrm{WF}\,}}_{[\mathfrak {M}]} u$ associated to ultradifferentiable classes given by weight matrices we refer to [11] and [10].

### Theorem 3.5

Let $P\in \mathcal {A}_{\rho ,\delta }^{d,d^\prime }(\Omega )$. Then the following holds:

1. *P* is $\{\mathfrak {M}\}$-hypoelliptic in Ω for any R-semiregular weight matrix $\mathfrak {M}$ such that $\textbf{G}^{\theta }\{\preceq \}\mathfrak {M}$.
2. *P* is $(\mathfrak {M})$-hypoelliptic in Ω for any B-semiregular weight matrix $\mathfrak {M}$ such that $\textbf{G}^{\theta }\{\lhd )\mathfrak {M}$.

### Proof

Following the arguments in the proof of [14, Theorem 3.1], see also [23, Theorem 3.26] and [4], one sees that for any $U\Subset \Omega$ there are operators $F,R:\mathcal {E}^\prime (U)\rightarrow \mathcal {D}^\prime (U)$ such that $FP={{\,\textrm{Id}\,}}+R$ in *U*. Moreover, the kernel of *R* is in $\mathcal {G}^{\theta }(U)$ whereas the kernel of *F*, which we also denote by *F*, satisfies ${{\,\mathrm{sing\, supp}\,}}_\theta F\subseteq \{ (x,x) \nonscript \, : \nonscript \, \mathopen  x\in U \}\subseteq U\times U$. Now, if $\mathfrak {M}$ is an R-semiregular weight matrix such that $\textbf{G}^\theta \{\preceq \}\mathfrak {M}$, then ${{\,\mathrm{sing\, supp}\,}}_{\{\mathfrak {M}\}}Fu\subseteq {{\,\mathrm{sing\, supp}\,}}_{\{\mathfrak {M}\}}u$ and $Ru\in \mathcal {E}^{\{ \mathfrak {M} \}} ( U )$ for all $u\in \mathcal {E}^\prime (U)$ by [10, Theorem 3.14]. Thence

$$\begin{aligned} {{\,\mathrm{sing\, supp}\,}}_{\{\mathfrak {M}\}} u={{\,\mathrm{sing\, supp}\,}}_{\{\mathfrak {M}\}} {(FP-R)u}\subseteq {{\,\mathrm{sing\, supp}\,}}_{\{\mathfrak {M}\}}{P}u\subseteq {{\,\mathrm{sing\, supp}\,}}_{\{\mathfrak {M}\}} u \end{aligned}$$

for all $u\in \mathcal {E}^\prime (U)$, which proves (1). The second part is proven completely analogously. □

Theorem 3.5 immediately implies Theorem 2.4(1),(2). The other parts of Theorem 2.4 also follow from Theorem 3.5 if we also consider the Remark below.

### Remark 3.6

If ω is a weight function then the weight matrix $\mathfrak {W}$ associated to ω consists of the weight sequences $\textbf{W}^\lambda$, λ>0, given by

$$\begin{aligned} W^{\lambda }_k=e^{\tfrac{1}{\lambda }\varphi ^*_\omega (\lambda k)}. \end{aligned}$$

Then $\mathfrak {W}$ has the following properties:

$$\begin{aligned}\begin{gathered} \;\,\forall \,\lambda>0:\quad W_{j+k}^\lambda \le W_{j}^{2\lambda }W_k^{2\lambda }\qquad \;\,\forall \,j,k\in \mathbb {N}_0, \\ \;\,\forall \,h>0 \;\,\exists \,A\ge 1 \;\,\forall \,\lambda \ge 0 \;\,\exists \,D\ge 1:\quad h^kW^\lambda _k\le DW^{A\lambda }_k\qquad \;\,\forall \,k\in \mathbb {N}_0. \end{gathered}\end{aligned}$$

In fact, $\mathcal {E}^{[ \omega ]} ( \Omega )=\mathcal {E}^{[ \mathfrak {W} ]} ( \Omega )$ as topological spaces, cf. [22]. Moreover it is well-known, that $\omega (t)=O(t^{1/s})$, s≥1 is equivalent to $\textbf{G}^s\{\preceq \}\mathfrak {W}$ and $\textbf{G}^s(\preceq )\mathfrak {W}$. In the same manner, $\omega (t)=o(t^{1/s})$ is equivalent to $\textbf{G}^s\{\lhd )\mathfrak {W}$.

## Ultradifferentiable scales and a general theorem of iterates

If *P* is a differential operator with analytic coefficients of order *d* and $\mathfrak {M}$ is a weight matrix satisfying (3.2) then the space $\mathcal {E}^{\{ \mathfrak {M} \}} \left( \Omega ; P \right)$ consists of those distributions $u\in \mathcal {D}^\prime (\Omega )$ such that $P^k u\in L^1_{loc}(\Omega )$ and for all $V\Subset \Omega$ there are $\textbf{M}\in \mathfrak {M}$ and C,h>0 such that

4.1 $$\begin{aligned} {\left\| {P^k{u}}\right\| }_{L^1(V)}\le Ch^k M_{dk}\qquad \;\,\forall \,k\in \mathbb {N}_0, \end{aligned}$$

whereas a distribution $u\in \mathcal {D}^\prime (\Omega )$ is an element of $\mathcal {E}^{ (\mathfrak {M}) } ( \Omega ; P )$ if $P^ku\in L^1_{loc}(\Omega )$ for all $k\in \mathbb {N}_0$ and for all $V\Subset \Omega$, every $\textbf{M}\in \mathfrak {M}$ and each h>0 there is a constant C>0 such that (4.1) holds.

If ω is a weight function with ω(t)=o(t) and $\mathfrak {W}$ is the weight matrix associated to ω then, in view of Remark 3.6, we have that $\mathcal {E}^{[ \omega ]} ( \Omega ; P )=\mathcal {E}^{[ \mathfrak {W} ]} ( \Omega ; P )$ for any differential operator *P* with analytic coefficients.

The rest of this paper is dedicated to the proof of Theorem 2.5 which will be in the same spirit as the proof of [12, Theorem 1.1]. For this we need to recall the definition of ultradifferentiable scales from [12, Subsection 4A]. However, in order to be able to use respective adapt the arguments of the proof of [2, Theoreme 1.2] we have to modify the definition of ultradifferentiable scales a little bit.

### Definition 4.1

Let Λ be a totally ordered set. A mapping $\zeta : \Lambda \times [0,+\infty )\longrightarrow [0,\infty )$ is said to be a generating function if for each $\lambda \in \Lambda$ the function $\zeta _\lambda =\zeta (\lambda ,.\,)$ is continuous and $\zeta _\lambda (t)/t$ is increasing on t≥1 and the following conditions are satisfied:

4.2 $$\begin{aligned} \zeta _\lambda (0)=0; \end{aligned}$$

4.3 $$\begin{aligned} \text {The sequence }\mathbb {N}\ni k\mapsto \zeta _{\lambda }(k)-\zeta _{\lambda }(k-1)\text { is increasing;} \end{aligned}$$

4.4 $$\begin{aligned} \lim _{t\rightarrow \infty }{\left( \frac{\zeta _{\lambda }(t)}{t}-\log t\right) }=\infty . \end{aligned}$$

We associate to a generating function a scale $(\textbf{M}^\lambda )_\lambda$ of weight sequences by setting

$$\begin{aligned} M_k^\lambda =\exp \left( \zeta (\lambda ,k)\right) . \end{aligned}$$

The matrix $\mathfrak {M}_\zeta =\mathfrak {M}=\{ \textbf{M}^\lambda \nonscript \, : \nonscript \, \mathopen  \lambda \in \Lambda \}$ is the weight matrix associated to the scale $(\textbf{M}^\lambda )_\lambda$. We see that $\mathfrak {M}_\zeta$ satisfies (3.2) due to (4.4).

We are going to need the following conditions:

4.5 $$\begin{aligned} \;\,\forall \,\lambda \in \Lambda \;\,\exists \,\lambda ^\prime {\in \Lambda } \;\,\exists \,\gamma >0: \quad \zeta _\lambda (2t)\le &   2\zeta _{\lambda ^\prime }(t)+\gamma t\qquad t\ge 1 \end{aligned}$$

4.6 $$\begin{aligned} \;\,\forall \,\lambda ^{\prime }\in \Lambda \;\,\exists \,\lambda {\in \Lambda } \;\,\exists \,\gamma >0: \quad \zeta _\lambda (2t)\le &   2\zeta _{\lambda ^\prime }(t)+\gamma t\qquad t\ge 1 \end{aligned}$$

Note that (4.5) (resp. (4.6)) implies that $\mathfrak {M}_{\zeta }$ satisfies (3.3) (resp. (3.4)), cf. [24]. In order that every weight sequence $\textbf{M}^\lambda$ is itself semiregular we need to require that

4.7 $$\begin{aligned} \;\,\forall \,\lambda \in \Lambda \;\,\exists \,\gamma >0:\quad \zeta _\lambda (p+1)-\zeta _\lambda (p)\le \gamma (p+1)\qquad p\in \mathbb {N}_0. \end{aligned}$$

Furthermore, we also consider the following conditions

4.8 $$\begin{aligned} \;\,\forall \,\alpha>0 \;\,\forall \,\lambda \in \Lambda \;\,\exists \,\lambda ^\prime \in \Lambda \;\,\exists \,\gamma >0:\quad \alpha \zeta _\lambda (t)\le &   \zeta _{\lambda ^\prime }(t)+\gamma (t+1)\qquad t\ge 1,\end{aligned}$$

4.9 $$\begin{aligned} \;\,\forall \,\alpha>0 \;\,\forall \,\lambda ^\prime \in \Lambda \;\,\exists \,\lambda \in \Lambda \;\,\exists \,\gamma >0:\quad \alpha \zeta _\lambda (t)\le &   \zeta _{\lambda ^\prime }(t)+\gamma (t+1)\qquad t\ge 1. \end{aligned}$$

Finally, for $\sigma \ge 1$ we say that the ultradifferentiable scale $(\textbf{M}^\lambda _\zeta )$ is superordinated to $\mathcal {G}^\sigma$ if

$$\begin{aligned} \;\,\forall \,\lambda \in \Lambda :\quad \lim _{t\rightarrow }\left( \frac{\zeta _\lambda (t)}{t}- \sigma \log t\right) =\infty . \end{aligned}$$

Clearly that means that $\{\textbf{G}^{\sigma }\}\{\lhd )\mathfrak {M}_\zeta$.

We are now able to begin with the proof of Theorem 2.5. To this end assume that $P\in \mathcal {A}_{\rho ,\delta }^{d,d^\prime }(\Omega )$. We are making use of the parametrices $A_N$ of the iterated operators $P^N$, $N\in \mathbb {N}$, constructed in [4]. Here we only briefly summarize the results we are going to need to analyze the regularity of ultradifferentiable vectors of *P*. The symbols $a_N$ of the operators $A_N$ have asymptotic expansions $a_N(x,\xi )\sim \sum _{j\ge 1} a_{N,j}(x,\xi )$ with the symbols $a_{N,j}$ being constructed in [4, II−1.2]. In fact, by [4, Equation (1.10)] the symbols $a_N$ are of the form

$$\begin{aligned} a_N(x,\xi ):=\sum _{j=0}^\infty \tilde{\chi }_j(\xi /\lambda )a_{N,j}(x,\xi ) \end{aligned}$$

for $x\in V\Subset \Omega$ and $\xi \in \mathbb {R}^n$. Here $\lambda \ge 1$ to be specified later on. Moreover ${\tilde{\chi }_j} (\eta )\in \mathcal {C}^\infty (\mathbb {R}^n)$ is a sequence such that $\tilde{\chi }_0(\eta )=0$ for $|\eta {|}\le L$ and $\tilde{\chi }_0(\eta ){=1}$ if $|\eta {|}\ge 2L$ and for j≥1 we have that

$$\begin{aligned} {\left\{ \begin{array}{ll} \tilde{\chi }_j(\eta )=0 &  |\eta {|}\le L\frac{j}{\rho -\delta };\\ \tilde{\chi }_j(\eta )=1 &  |\eta {|}\ge 2L\frac{j}{\rho -\delta };\\ |D^\alpha \tilde{\chi }_j(\eta ){|}\le C^{{|\alpha |}+1}j^{{|\alpha |}(1- 1/(\rho -\delta )} &  {|\alpha |}\le j \end{array}\right. } \end{aligned}$$

for a constant C>0 independent of *j*, cf. [4, pp. 57–58].

According to [4, Corollaire 2.7] there are λ>0, ε>0 and C>0 such that for all $\xi \in \mathbb {R}^n$, ${|\xi |}\ge C$, all $N\in \mathbb {N}$, $\psi \in \mathcal {C}^\infty _0(\overline{V})$ and all $u\in \mathcal {D}^\prime (\Omega )$ with $P^ku\in L^1_{loc}(\Omega )$ for $k\in \{1,\dotsc ,N\}$ we have that

$$\begin{aligned} \widehat{\psi u}(\xi )=\mathcal {F}\bigl (A_N\psi P^Nu\bigr )(\xi )+ \sum _{k=1}^N\mathcal {F}\bigl (S_k\psi P^{k-1}u\bigr )(\xi )+\sum _{k=1}^N\mathcal {F}\bigl ( A_k[P,\psi ]P^{k-1}u\bigr )(\xi ) \end{aligned}$$

where $S_k$ are error terms (defined in [4, p. 63]) satisfying

$$\begin{aligned} |\widehat{S_kv}(\xi ){|}\le C^{k+1} |\xi {|}^{-d^\prime k} e^{\varepsilon |\xi {|}^{\rho -\delta }}{\left\| {v}\right\| }_{L^1(V)} \end{aligned}$$

for $$v\in L^k(V)$$ and $$|\xi {|}\ge 2\lambda L$$.

Now let $x_0\in V\Subset \Omega$ and we fix also $\psi \in \mathcal {C}^\infty _0(V)$ equal to 1 near $x_0$. We choose also a sequence $\varphi _N\in \mathcal {D}(V)$ such that $\varphi _N=1$ near $x_0$ and $\varphi _N\psi =\varphi _N$ and for all $\beta \in \mathbb {N}^n_0$ there is a constant $C_\beta$ so that

$$\begin{aligned} |D^{\alpha +\beta }\varphi _N(x){|}\le C_\beta ^{{|\alpha |}+1} N^{{|\alpha |}},\qquad x\in V,\; {|\alpha |}\le 2d^\prime N \end{aligned}$$

for all N≥1. Moreover, we set $\chi _N(x)=\tilde{\chi }_{qN}(x/2\lambda )$ where $q=[2d^\prime /\rho ]+2$. Then we have

4.10 $$\begin{aligned} \widehat{\varphi _N u}= \widehat{\varphi _N \psi u}&= \hat{\varphi }_N*(1-\chi _N)\widehat{\psi u}+\hat{\varphi }_N*\chi _N \mathcal {F}(A_N \psi P^N u)\nonumber \\&\quad +\sum _{k=1}^N \hat{\varphi }_N*\chi _N \mathcal {F}\left( S_k \psi P^{k-1}u\right) +\sum _{k=1}^N \hat{\varphi }_N*\chi _N\mathcal {F}\left( A_k[P,\psi ]P^{k-1}u\right) \end{aligned}$$

for $u\in \mathcal {D}^\prime (\Omega )$ with $P^ku\in L^1_{loc}(\Omega )$ for all $k\in \mathbb {N}_0$.

We need to estimate the right-hand side of (4.10) first for $u\in \mathcal {D}^\prime (\Omega )$ satisfying the following estimates: There are a weight sequence $\textbf{M}$ and constants C,h>0 such that

$$\begin{aligned} {\left\| {P^Nu}\right\| }_{L^{1}(V)}\le Ch^N M_{dN},\qquad \;\,\forall \,N\in \mathbb {N}_0{,} \end{aligned}$$

and we recall from above that $\Lambda _k=\root k \of {M_k}$ denotes the sequence of quotients associated to $\textbf{M}$.

By the proof of [4, Corollary 2.9] we obtain that there is a constant $C_1>0$ such that for ${|\xi |}> C_1$ and $1\le j\le k$ we have that

$$\begin{aligned}&|\hat{\varphi }_k*\chi _N \mathcal {F}\left( S_k \psi P^{k-1}u\right) +\sum _{k=1}^N \hat{\varphi }_N*\chi _N\mathcal {F}\left( A_k[P,\psi ]P^{k-1}u\right) {|}\\&\quad \le C_1^{N+1} N^{\tfrac{d^\prime k}{\rho -\delta }} h^k M_{d(k-1)} {|\xi |}^{-d^\prime N-d^\prime k+d-1}. \end{aligned}$$

On the other hand, observe that

$$\begin{aligned} \sum _{k=1}^N \frac{M_{d(k-1)}}{{|\xi |}^{d^\prime k}} \le \sum _{k=1}^N \left( \frac{\Lambda _{dN}}{{|\xi |}^{d^\prime /d}}\right) ^{dk} \le \sum _{k=1}^\infty \left( \frac{1}{2^d}\right) ^j<\infty \end{aligned}$$

if ${|\xi |}^{d^\prime /d}\ge 2 \Lambda _{dk}$. Thus we have proven the following statement.

### Lemma 4.2

There exists a constant $C_2>0$ such that

$$\begin{aligned} |\sum _{k=1}^N \hat{\varphi }_N*\chi _N\mathcal {F}\left( A_k[P,\psi ]P^{k-1}u\right) {|}\le C_2^{N+1}N^{\tfrac{d^\prime N}{\rho -\delta }} {|\xi |}^{-d^\prime N+d-1} \end{aligned}$$

for $${|\xi |}\ge 2C_2 \Lambda _{dN}^{d/d^\prime }$$.

Regarding the third term we have by the proof of [4, Proposition 2.10] that there are constants $C_3,C_4>0$ such that

$$\begin{aligned} |\hat{\varphi }_N*\chi _N\mathcal {F}\left( S_k\psi P^{k-1} u\right) {|}\le C_4^{N+1} N^{\tfrac{d^\prime N}{\rho -\delta }} h^k M_{dk} {|\xi |}^{-d^\prime k^N-d^\prime k +n+1} \end{aligned}$$

for ${|\xi |}\ge C_3$ and j=1,⋯,k and $k\in \mathbb {N}$. As above we thus get:

### Lemma 4.3

There exists a constant $C_5>0$ such that

$$\begin{aligned} |\sum _{k=1}^N\hat{\varphi }^\prime _N*\chi _N\mathcal {F}\bigl (S_k\psi P^{k-1}u\bigr )(\xi ){|}\le C_5^{N+1}N^{\frac{d^\prime N}{\rho -\delta }} {|\xi |}\le C_5^{N+1} (d^\prime N)^{\frac{d^\prime N}{\rho -\delta }} {|\xi |}^{-d^\prime N+n+1} \end{aligned}$$

for $${|\xi |}\ge C_5\Lambda _{dN}^{d/d^\prime }$$.

For the first term, we observe that by [4, Proposition 2.12] there is a constant $C_7>0$ such that for all $N\in \mathbb {N}$ and ${|\xi |}\ge C_7$ we have that

$$\begin{aligned} |\hat{\varphi }_N*(1-\chi _N)\widehat{\psi u}{|}\le C_7^{N+1} \left( d^\prime N \right) ^{\frac{d^\prime N}{\rho -\delta }}{|\xi |}^{-d^\prime N+n+1}{\left\| {u}\right\| }_{L^1(V)}. \end{aligned}$$

Finally, regarding the second term the proof of [4, Proposition 2.11] gives that

$$\begin{aligned} |\hat{\varphi }_N*\chi _N\mathcal {F}(A_N\psi P^Nu)(\xi ){|}\le C_6^{N+1} h^N M_{dN}{|\xi |}^{-d^\prime N+n+1}\qquad {|\xi |}\ge C_6. \end{aligned}$$

Assume now that $\textbf{M}=\textbf{M}^\lambda _\zeta$ for some generating function satisfying (4.5). Iterating (4.5) shows that for each $\lambda \in \Lambda$ and α>0 there are $\lambda ^\prime \in \Lambda$ and γ>0 such that

$$\begin{aligned} \frac{\zeta _\lambda (\alpha t)}{\alpha t}\le \frac{\zeta _\lambda \bigl (2^\nu t\bigr )}{2^\nu t}\le \frac{\zeta _\lambda (t)}{t}+\gamma ,\qquad t\ge 1 \end{aligned}$$

where $\nu \in \mathbb {N}$ is the smallest integer such that $\alpha \le 2^\nu$. Thus we obtain

$$\begin{aligned}&|\hat{\varphi }_N*\chi _N\mathcal {F}(A_N\psi P^Nu)(\xi ){|}\\&\quad \le C_7^{N+1} h^N e^{\lambda dN}\exp \biggl (\frac{d}{d^\prime }\zeta _{\lambda ^\prime }(d^\prime N)\biggr ){|\xi |}^{-d^\prime N+n+1}\qquad {|\xi |}\ge C_7. \end{aligned}$$

Summarizing we have proven the following fact: if $(\textbf{M}^\lambda _\zeta )$ is an ultradifferentiable scale superordinate to $\textbf{G}^\theta$ such that (4.5) holds and $u\in \mathcal {E}^{\{ \textbf{M}^\lambda \}} \left( \Omega ; P \right)$ (resp. $u\in \mathcal {E}^{ (\textbf{M}^\lambda ) } ( \Omega ; P )$ then there exist $\lambda ^\prime \in \Lambda$ and a constant c>0 and some $C_0,h_0>0$ (resp. for each $h_0>0$ there is a constant $C_0$) such that

$$\begin{aligned} |\widehat{\varphi _N u}(\xi ){|}\le C_0 h_0^N \exp \left( \frac{d}{d^\prime }\zeta _{\lambda ^\prime }(d^\prime N)\right) {|\xi |}^{-d^\prime N+d+n} \end{aligned}$$

for ${|\xi |}\ge c\exp (d/(d^\prime )^2\zeta _\lambda (d^\prime N))$ and N≥1. Since the sequence $(\varphi _N u)_N$ is bounded in $\mathcal {E}^\prime (\Omega )$ the Banach-Steinhaus theorem implies that there is a constant $\mu \ge 0$ such that that there is a constant $C_9>0$ such that

$$\begin{aligned} |\widehat{\varphi _Nu}{|}\le C_9^{N+1} \bigl (1+{|\xi |}\bigr )^{\mu }. \end{aligned}$$

Thence, in fact, there are $C_0,h_0>0$ (resp. for every $h_0>0$ there exists $C_0>0$) such that

$$\begin{aligned} {|\xi |}^{d^\prime N-d-n-\mu }|\widehat{\varphi _N u}(\xi ){|} \le C_0 h_0^N \exp \left( \frac{d}{d^\prime } \zeta _{\lambda ^{\prime }}(d^\prime N)\right) \end{aligned}$$

for $$N\in \mathbb {N}$$ and $$\xi \in \mathbb {R}^n$$.

In order to continue for $N\in \mathbb {N}$ we set

$$\begin{aligned} v_N=\varphi _{\widehat{N}}u,\qquad \widehat{N}=\bigg \lceil \frac{N+d+n+\mu }{d^\prime }\bigg \rceil , \end{aligned}$$

where $\lceil \rho \rceil$ is the smallest integer larger than ρ. In particular note that $N+d+n+\mu \le d^\prime \widehat{N}\le N+d+n+\mu +\tilde{d}$, where $\tilde{d}=\lceil d^\prime \rceil$. By the above estimates we obtain

$$\begin{aligned} {\begin{matrix} {|\xi |}^N|\hat{v}_N{|}& \le C_0h_0^N \exp \left( \frac{d}{d^\prime }\zeta _{\lambda ^\prime }(d^\prime N)\right) {|\xi |}^{N-d^\prime \widehat{N}+d+n+\mu }\\ & \le C_0 h_0^N \exp \left( \frac{d}{d^\prime }\zeta _{\lambda ^\prime }(N+d+n+\mu +\tilde{d})\right) {|\xi |}^{N-N-d-n-\mu +d+n+\mu }\\ \end{matrix}} \end{aligned}$$

for ${|\xi |}\ge 1$. If (4.8) holds then we have that there exists $\tilde{\lambda }\in \Lambda$ such that there are $C_0,h_0>0$ (resp. for each $h_0>0$ there is $C_0>0$) so that

$$\begin{aligned} {|\xi |}^N|\hat{v}_N{|}\le C_0h_0^N \exp \left( \zeta _{\tilde{\lambda }} (N+d+n+\mu +\tilde{d})\right) \qquad \;\,\forall \,{|\xi |}\ge 1,\;\;\,\forall \,N\in \mathbb {N}, \end{aligned}$$

and therefore, if (4.7) holds then there are $C_0,h_0>0$ (resp. for every $h_0$ there is $C_0>0$) such that

$$\begin{aligned} {|\xi |}^N|\hat{v}_N{|}\le C_0h_0^N \exp \left( \zeta _{\tilde{\lambda }} (N)\right) \qquad \;\,\forall \,{|\xi |}\ge 1,\;\;\,\forall \,N\in \mathbb {N}. \end{aligned}$$

Thence using Theorem 3.4 we obtain that $x_0\notin {{\,\mathrm{sing\, supp}\,}}_{{[}\textbf{M}^{\tilde{\lambda }}{]}} u$ and since $x_0\in V\Subset \Omega$ have been chosen arbitrarily we have proven the following theorem.

### Theorem 4.4

Let $P\in \mathcal {A}^{d,d^\prime }_{\rho ,\delta }(\Omega )$ and $(\textbf{M}^\lambda _\zeta )$ be an ultradifferentiable scale superordinate to $\textbf{G}^\theta$, $\theta =\tfrac{1}{\rho -\delta }$. If the generating function ζ additionally satisfies (4.5), (4.7) and (4.8) then for any λ there is some $\tilde{\lambda }$ such that

$$\begin{aligned} \mathcal {E}^{[ \textbf{M}^\lambda ]} ( \Omega ; P )\subseteq \mathcal {E}^{[ \textbf{M}^{\tilde{\lambda }} ]} ( \Omega ). \end{aligned}$$

In fact, since our arguments are completely local, we can easily see that also the following statement is true.

### Theorem 4.5

Let $P\in \mathcal {A}^{d,d^\prime }_{\rho ,\delta }(\Omega )$ and $(\textbf{M}^\lambda _\zeta )$ be an ultradifferentiable scale superordinate to $\textbf{G}^\theta$, $\theta =\tfrac{1}{\rho -\delta }$.

1. If (4.5) and (4.8) hold then $\mathcal {E}^{\{ \mathfrak {M}_\zeta \}} \left( \Omega ; P \right) =\mathcal {E}^{\{ \mathfrak {M}_\zeta \}} ( \Omega )$.
2. If (4.6) and (4.9) hold then $\mathcal {E}^{ (\mathfrak {M}_\zeta ) } ( \Omega ; P )=\mathcal {E}^{( \mathfrak {M}_\zeta )} ( \Omega )$.

Theorem 2.5 follows now from Theorem 4.5 and the following Remark.

### Remark 4.6

Let ω be a weight function. According to [12, Section 5A] $\zeta _\omega (t,\lambda )=\frac{1}{\lambda }\varphi ^*_\omega (\lambda t)$ is a generating function. The convexity of $\varphi ^*$ implies that

$$\begin{aligned} \zeta _\omega (2t,\lambda )\le 2\zeta _\omega (t,2\lambda ). \end{aligned}$$

Hence both (4.5) and (4.6) are satisfied. Moreover, by [17, (A.2)] condition (Ξ) implies that

$$\begin{aligned} \;\,\exists \,C,H> 0\;\,\forall \,x>0:\quad C\varphi _\omega ^*\left( \frac{x}{C}\right) \le \varphi ^*_\omega \left( \frac{x}{2}\right) +(\log H)x+C. \end{aligned}$$

Arguing as in [17, p. 2172] we see that we can conclude that (Ξ) gives that

$$\begin{aligned} \;\,\exists \,A\ge 1\;\,\forall \,\lambda>0\;\,\exists \,\gamma>0:\quad \zeta _\omega (2t,\lambda )\le \zeta _\omega (t,A\lambda )+\gamma (t+1)\qquad \;\,\forall \,t>0. \end{aligned}$$

Since we have that $\varphi ^*(s)+\varphi ^*(t)\le \varphi ^*(s+t)$, cf. [22], we obtain in fact

$$\begin{aligned} \;\,\exists \,A\ge 1\;\,\forall \,\lambda>0\;\,\exists \,\gamma >0:\qquad 2\zeta _\omega (t,\lambda )\le \zeta _\omega (t,A\lambda )+\gamma (t+1)\qquad t\ge 0. \end{aligned}$$

Thence, if ω satisfies (Ξ) then both (4.8) and (4.9) hold for the generating function $\zeta _\omega$.

Moreover, by [13, Remark 2.8(3)] we obtain that if ω is a weight function such that (Ξ) holds then $\omega (t)=o(t^\gamma )$ for any 0<γ<1. Now according to [25, Corollary 5.10] the fact $\omega (t)=o(t^\gamma )$, 0<γ<1, is equivalent to the fact that $\textbf{G}^\sigma \lhd \textbf{W}^\lambda$ for all λ>0, where $\sigma =1/\gamma$. That means that, if (Ξ) holds for ω then

4.11 $$\begin{aligned} \;\,\forall \,\sigma>1\;\,\forall \,\lambda >0 \qquad \lim _{\mathbb {N}\ni k\rightarrow \infty }\frac{k^\sigma }{\exp \left( \frac{\varphi ^*_\omega (\lambda k)}{\lambda k}\right) }=0{.} \end{aligned}$$

We want to replace $k\in \mathbb {N}$ in the limit with t>0. To this end observe that both $t^\sigma$, σ>1, and the function $t\mapsto {\tfrac{1}{t}}\varphi ^*_\omega (t)$ are increasing by [6]. Thence we obtain for t∈[k,k+1], $k\in \mathbb {N}$, and $s=\lceil \sigma \rceil$ that

$$\begin{aligned} \frac{t^\sigma }{\exp \left( \frac{\varphi ^*_\omega (\lambda t)}{\lambda t}\right) } \le \frac{(k+1)^{s}}{\exp \left( \frac{\varphi ^*_\omega (\lambda k)}{\lambda k}\right) } =\sum _{\ell ={0}}^{{s}} \left( {\begin{array}{c}{s}\\ \ell \end{array}}\right) \frac{{k}^{{s}-\ell }}{\exp \left( \frac{\varphi ^*_\omega (\lambda k)}{\lambda k}\right) }\xrightarrow []{\quad {k}\rightarrow \infty \quad }0 \end{aligned}$$

by (4.11). Therefore we have proven that if ω is a weight function satisfying (Ξ) then the ultradifferentiable scale generated by $\zeta _\omega (t,\lambda )={\tfrac{1}{\lambda }}\varphi _\omega ^*(\lambda t)$ is superordinate to $\textbf{G}^\sigma$ for all $\sigma \ge 1$.

## Data Availability

No datasets were generated or analysed during the writing of this article.
